# STOPPIT Baby Follow-Up Study: The Effect of Prophylactic Progesterone in Twin Pregnancy on Childhood Outcome

**DOI:** 10.1371/journal.pone.0122341

**Published:** 2015-04-16

**Authors:** Helen Christine McNamara, Rachael Wood, James Chalmers, Neil Marlow, John Norrie, Graeme MacLennan, Gladys McPherson, Charles Boachie, Jane Elizabeth Norman

**Affiliations:** 1 Monash University, Melbourne, Australia; 2 Information Services Division, NHS National Services Scotland, Edinburgh, United Kingdom; 3 Department of Neonatal Medicine, Institute for Women's Health, University College London, London, United Kingdom; 4 Centre for Healthcare Randomised Trials (CHaRT), Health Services Research Unit, University of Aberdeen, Aberdeen, United Kingdom; 5 Robertson Centre for Biostatistics, University of Glasgow, Glasgow, United Kingdom; 6 Tommy’s Centre for Maternal and Fetal Health, University of Edinburgh MRC Centre for Reproductive Health, The Queen’s Medical Research Institute, Edinburgh, United Kingdom; Shanghai Jiaotong University School of Medicine, CHINA

## Abstract

**Objectives:**

To determine the long-term effects of *in utero* progesterone exposure in twin children.

**Methods:**

This study evaluated the health and developmental outcomes of all surviving children born to mothers who participated in a double-blind, placebo-controlled trial of progesterone given for the prevention of preterm birth in twin pregnancies (STOPPIT, ISRCTN35782581). Follow-up was performed via record linkage and two parent-completed validated questionnaires, the *Child Development Inventory* and the *Health Utilities Index*.

**Results:**

Record linkage was successfully performed on at least one record in 759/781 (97%) children eligible for follow-up. There were no differences between progesterone-exposed and placebo-exposed twins with respect to incidence of death, congenital anomalies and hospitalisation, nor on routine national child health assessments. Questionnaire responses were received for 324/738 (44%) children. The mean age at questionnaire follow-up was 55.5 months. Delay in at least one developmental domain on the *Child Development Inventory* was observed in 107/324 (33%) children, with no evidence of difference between progesterone-exposed and placebo-exposed twins. There was no evidence of difference between the progesterone and placebo groups in global health status assessed using the *Health Utilities Index*: 89% of children were rated as having ‘excellent’ health and a further 8% as having ‘very good’ health.

**Conclusions:**

In this cohort of twin children there was no evidence of a detrimental or beneficial impact on health and developmental outcomes at three to six years of age due to *in utero* exposure to progesterone.

## Introduction

Preterm birth, defined as birth prior to 37 weeks’ estimated gestation, is a leading cause of perinatal mortality and short-term and long-term morbidity. Twin pregnancies contribute disproportionately to preterm birth.

Progesterone, administered either as intramuscular 17 α-hydroxyprogesterone caproate or vaginal progesterone, has been demonstrated to reduce the rate of preterm birth in women with high-risk singleton pregnancies [1–7]. In contrast, we and others have shown that neither progesterone nor 17 α-hydroxyprogesterone caproate prevents preterm birth in multiple pregnancies [8–13]. Use of progesterone for prevention of preterm birth in women with a previous preterm birth and/or with a short cervix is becoming widespread [14]. In 2011, the USA Food and Drug Administration approved use of 17 α-hydroxyprogesterone caproate for the prevention of preterm birth in women at risk because of previous spontaneous preterm birth [15].

Previous studies have demonstrated that agents given to pregnant women with the aim of improving pregnancy outcomes can have unexpected effects on children which may not be apparent at birth but harmful long-term [16]. Hence, long-term follow-up data on children exposed to progesterone are needed.

There is emerging but limited evidence on the long-term effects of *in utero* exposure to progesterone when given for the prevention of preterm birth. Follow-up of 274 singleton children, born to mothers participating in a randomised placebo-controlled trial of 17 α-hydroxyprogesterone caproate in high-risk singleton pregnancies, demonstrated no difference in health status, physical examination, or mean score on the *Ages and Stages Questionnaire* at four years of age despite prolongation of pregnancy [17]. Follow-up of 991 children born to mothers with twin pregnancies enrolled in the PREDICT randomised control trial comparing progesterone with placebo revealed no difference in mean score on the *Ages and Stages Questionnaire* at six months and at 18 months of age. Progesterone had no effect on duration of pregnancy in the PREDICT trial [12].

The aim of our study was to determine the potential adverse and/or beneficial effects of prophylactic *in utero* progesterone on health and developmental outcomes of children at three to six years of age. We evaluated a cohort of twin pregnancies in which progesterone had no effect on short-term (obstetric and neonatal) outcomes, thus allowing any direct effects of progesterone to be determined.

## Methods

This study evaluated outcomes of twin children born to mothers who participated in the STOPPIT trial, a randomised, double-blind, placebo-controlled trial of progesterone given for the prevention of preterm birth to women with twin pregnancy.

Briefly, the STOPPIT trial (registered clinical trial, ISRCTN35782581), conducted at nine National Health Service (NHS) hospitals across the United Kingdom, randomised women to receive either progesterone gel or placebo gel daily from 24 weeks’ gestation for an average period of ten weeks. In the original trial, outcome assessment was limited to obstetric and neonatal outcomes. These results have previously been reported: there were no differences in the primary outcome of intrauterine death or preterm delivery prior to 34 weeks and 0 days of gestation, nor any differences in neonatal outcomes between the progesterone and placebo groups [10]. All participating women were informed of the possibility of follow-up of their children and were given the opportunity to withdraw consent for future contact from the research team if they wished.

We aimed to include all children born to mothers resident in Scotland who had been recruited to the STOPPIT trial, and for whom routine health data could therefore be accessed through Information Services Division, NHS Scotland. Given that such routine health data were available for twins resident in Scotland, mothers who were resident in England at the time of the trial, and all those who emigrated out of a Scottish Health Board after the birth of their babies were not eligible for follow-up. Mothers who were lost to follow-up, and those for whom there were insufficient data for tracing were excluded from follow-up. Mothers who withdrew consent for questionnaire follow-up and those who had experienced the death of one or more twins were also excluded.

The personal identifiers of mothers of eligible children were used to identify mothers’ records on the Community Health Index (CHI) database and thus obtain their up to date contact details to enable questionnaire distribution. The CHI database contains a record of all patients registered with a General Practitioner in Scotland.

The personal identifiers of mothers were also used to identify the CHI numbers of children via linkage to statutory birth registration records. CHI numbers of children are documented on all routine health records in Scotland, and hence record linkage enabled follow-up of twins’ outcomes through examination of routine health data. In addition, statutory death registration records were searched using children’s personal identifiers to identify any additional deaths of twins for whom the CHI numbers were unknown.

Records for twins with known CHI numbers were identified on the national child health programme information systems *Health Visitor First Visit*, *Six to Eight Week Check*, *Primary-1 Screening*, *Neonatal Hearing Screening* and *Preschool Vision Screening* records. Subsequent to April 2005, child health programme records included a Health Plan Indicator assigned to indicate a child’s level of need for ongoing support to achieve good health and developmental outcomes.

Twin records were also identified on the hospital outpatient attendance (*Scottish Morbidity Record 00—SMR00*) and inpatient and day case discharge (*SMR01*) datasets. For all twins with hospital attendances, primary diagnoses at time of discharge (coded via World Health Organisation International Classification of Disease, 10^th^ edition (ICD-10)[18]) were examined. Those with outpatient or inpatient entries with diagnoses ICD-10 codes Q00-Q99 (Chapter XVII: Congenital malformations, deformations and chromosomal abnormalities) were defined as having a congenital abnormality.

Twins in the progesterone and placebo groups were heterogeneous with respect to age, sex, and gestational age at birth. Accordingly, measures of height, weight and head circumference were converted into z-scores (standard deviations) using freely available LMS software [19] and UK 1990 growth reference data [20].

We selected two validated parent-completed questionnaires, the *Health Utilities Index* (HUI) [21] and the *Child Development Inventory* (CDI) [22] from available paediatric developmental assessment tools, purchasing licenses for the use and exact reproduction of each questionnaire. Mothers were invited by letter to participate in questionnaire follow-up. All letters were accompanied by a demographic data questionnaire, an information sheet and a written consent form. Where telephone numbers of mothers were available, a courtesy call was made to ensure questionnaires had been received. Questionnaires were mailed out in 2011 and again in 2012. In the unusual event that a mother replied twice, data from the most recent questionnaire were used. Mothers completed and returned all questionnaires and investigators calculated aggregate scores prior to any unmasking. Thereafter, treatment allocation was revealed to four mothers at their request.

We compared baseline demographic characteristics including maternal age at randomisation, household index of multiple deprivation (quintile), gestation and chorionicity, and the age of the children at the time of questionnaire completion in the responding and non-responding groups.

We summarised all baseline characteristics and outcomes using means (standard deviation), medians (25^th^ and 75^th^ centiles) or frequencies and percentages where relevant. Outcomes were analysed using generalised linear models (linear or logistic regression) where appropriate, adjusting for the clustering at the level of the mother. For other outcomes, Fisher’s exact test or a comparison of medians was used. Where possible, all effect sizes have 95% percent confidence intervals. All analyses were conducted using SAS version 9.2 (SAS Institute, Cary, North Carolina). There was no imputation of missing data and all analyses were as randomised.

Ethics approval was granted by the South East Scotland Research Committee 02 (reference number 10/S1102/70).

All women recruited to the original STOPPIT trial had been informed of the possibility of direct and record-based follow-up of their babies prior to providing written informed consent for participation. At the conclusion of the trial, women were reminded of the intention to complete a follow-up study in a newsletter and were given the opportunity to withdraw consent for further contact from the research team.

At the time of the follow-up study, Community Health Index Advisory Group (CHIAG) and Privacy Advisory Committee (PAC) approvals were obtained in order to update contact details of mothers of children eligible for follow-up, identify CHI numbers of eligible children, and perform anonymised record linkage of children’s CHI numbers to national child health records so that health data could be obtained. Parents were contacted and gave additional written informed consent for the completion of questionnaires.

## Results

**Fig 1:**
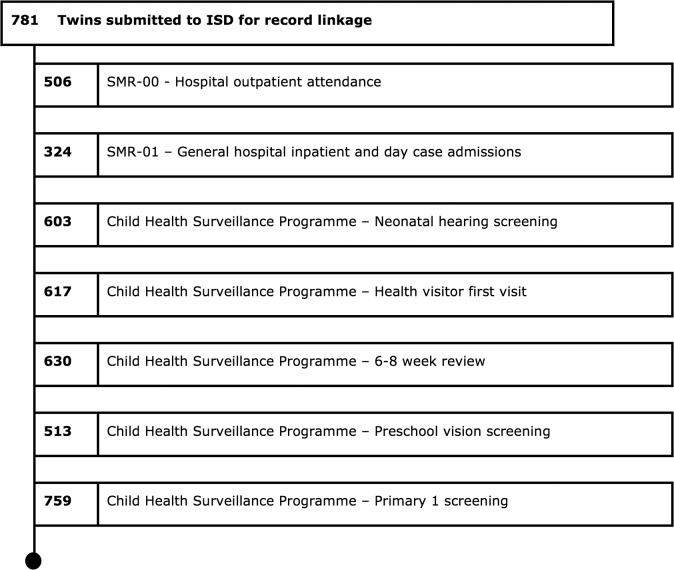
Flow of twins through record linkage at ISD.

Of the 500 women in the original STOPPIT trial, 68 were excluded from attempted record linkage as they lived in England (and would therefore not have relevant health records in Scotland), leaving 432 for whom record linkage was attempted. A further six women were lost to follow-up at the end of the original trial and ten could not be identified on the CHI database. Therefore, 416 women were potentially eligible to be sent a questionnaire and had current contact details available. Linkage to the children’s CHI numbers was performed in March 2013. CHI numbers of one child (in seven women) and both children (in 22 women) could not be identified, leaving 781 children whose CHI numbers were submitted for linkage to national child health records at ISD (386 in the progesterone group and 395 in the placebo group). Record linkage was successfully performed on at least one record in 759/781 (97%) children. The numbers of children for whom records were linked in each group are shown in Fig 1.

Stillbirth, neonatal or paediatric death was identified in 26 twins in the study cohort: 15 twins in the progesterone group and 11 twins in the placebo group. Two deaths were identified as having occurred since the findings of the original STOPPIT trial were reported. Deaths, congenital malformations, and health service utilisation were similar in the progesterone and control groups.

The rate of congenital malformations, identified using data pertaining to primary diagnoses in children with hospital attendances, was 4% in both the progesterone and placebo groups (Table 1).

**Table 1:** Health service use and rate of congenital malformations identified in hospital.

	Progesterone	Placebo	
	n/N children (%)	n/N children (%)	Effect size (95% CI), p-value
**Hospital outpatient attendance record (SMR-00)**			
Number of children with any attendance	246/386 (64)	260/395 (66)	0.91 (0.64–1.28), 0.58
Median number of visits per child*	0 [0, 1]	0 [0, 1]	*p* = 0.65
**General hospital inpatient record (SMR-01)**			
Number of children with any admission	159/386 (41)	165/395 (42)	0.97 (0.71–1.33), 0.87
Median cumulative length of stay per admission (days)*	39 [10, 62]	31 [10, 59]	p = 0.26**
**Congenital malformations (SMR-01)**			
Number of children with any congenital malformation	17/386 (4)	17/395 (4)	1.04 (0.49–1.21), 0.92

*Data presented as median [IQR]

**Two-sided p value from Wilcoxon Rank Sum test

Health service utilisation, including outpatient hospital attendances and inpatient hospitalisations, was similar for twins in the progesterone and placebo groups. For those who were hospitalised, the median (interquartile range) cumulative length of stay was 39 (10, 62) days in the progesterone group and 31 (10, 59) days in the placebo group (p = 0.26).

Child Health Surveillance Programme data revealed no differences in terms of developmental screening between twins in the progesterone and placebo groups (Table 2). There were no differences in the incidence of developmental concern raised by the parent at the *Health Visitor First Visit* nor in developmental assessment at the *Six to Eight Week Check*. Height and weight data collected at the *Primary-1 Screening* demonstrated no evidence that progesterone exposure *in utero* had an important effect on growth in childhood. Both progesterone and placebo exposed children were near to but less than the 50^th^ centile for weight and height. Although height centile was marginally significantly lower in the progesterone group compared with the placebo group, there were no differences in mean height. Hence, the clinical relevance of the height centile difference is likely to be limited.

**Table 2:** Child Health Surveillance Programme.

	**Progesterone**	**Placebo**	
**Health Visitor First Visit** (0–10 days of life)	**n/N children (%)**	**n/N children (%)**	**Effect size (95% CI), p-value**
Parental concern*			
Number of children with any concern identified	53/307 (17)	77/311 (25)	0.65 (0.40–1.07), 0.09
**Health Plan Indicator**			
Core	183/307 (60)	191/311 (61)	0.043^†^
Additional	21/307 (7)	26/311 (8)	
Intensive	23/307 (7)	8/311 (3)	
PH4P^#^ or unknown	80/307 (26)	86/311 (28)	
**6–8 Week Check** (6–8 weeks of life)			
Developmental assessment**			
Number of children with any domains identified as doubtful/uncertain or abnormal	8/310 (3)	15/320 (5)	0.55 (0.21–1.46), 0.23
** Health Plan Indicator**			
Core	182/310 (59)	176/320 (55)	0.47^†^
Additional	48/310 (15)	55/320 (17)	
Intensive	10/310 (3)	6/320 (2)	
PH4P^#^ or unknown	70/310 (23)	83/320 (26)	
**Primary 1 Screening** (4.5–5.5 years)	**Mean (SD), *n***	**Mean (SD), *n***	**Mean difference [95% CI], p-value**
** Measurement and growth**			
Height (cm)	111 (5), 165	112 (5), 189	-0.6 [-2.1, 0.8], 0.41
Weight (kg)	19.4 (3.2), 165	19.7 (2.8), 189	0.2 [-1.0, 1.4], 0.74
BMI	15.6 (2), 165	15.6 (1.5), 189	0.2 [-0.3, 0.4], 0.64
Height centile	42.1 (28), 165	48.5 (28.8), 189	-6.4 [-12.3, -0.4], 0.04
Weight centile	43.5 (29.7), 165	48.6 (29.2), 189	-5.2 [-11.3, 1.0], 0.10
** Health Plan Indicator**			
Core	236/371 (64)	264/388 (68)	0.43^†^
Additional	50/371 (13)	39/388 (10)	
Intensive	3/371 (1)	2/388 (1)	
PH4P^#^ or unknown	82/371 (22)	83/388 (21)	

* The health visitor records parental concerns relating to the child’s feeding, illness, crying, appearance, weight and sleep.

** The health visitor assesses the following developmental domains: gross motor; hearing and communication; and vision and social awareness. Development is assessed to be normal; abnormal; doubtful/uncertain; or not assessed/incomplete.

^#^PH4P (Pre-Hall 4 Programme): The Health for All Children screening and surveillance programme was introduced in Scotland from April 2005. The PH4P utilises the Health Plan Indicator. Children assessed prior to the introduction of the PH4P at local health care providers were not classified using this system

^†^ P-value for chi-square test of association.

There were no differences in the outcomes of sensory screening between the progesterone and placebo groups (Table 3).

**Table 3:** Sensory Screening.

	Progesterone	Placebo	
	n/N children (%)	n/N children (%)	Effect size (95% CI), p-value
Neonatal hearing screening *			
Either ear fail	41/302 (14)	31/301 (10)	1.38 (0.81–2.35), 0.23
Preschool vision screening **			
Either eye fail	70/250 (28)	71/263 (27)	1.04 (0.67–1.64), 0.85

*Screeners record the outcome of the first screen of each ear as pass, fail/refer, or not done/incomplete.

**Preschool vision screening includes an assessment of visual acuity and ocular movement. The outcome of screening for each eye is recorded as pass, refer or ongoing follow-up (both indicating failing screening), or recall (indicating screening could not be satisfactorily completed).

**Fig 2:**
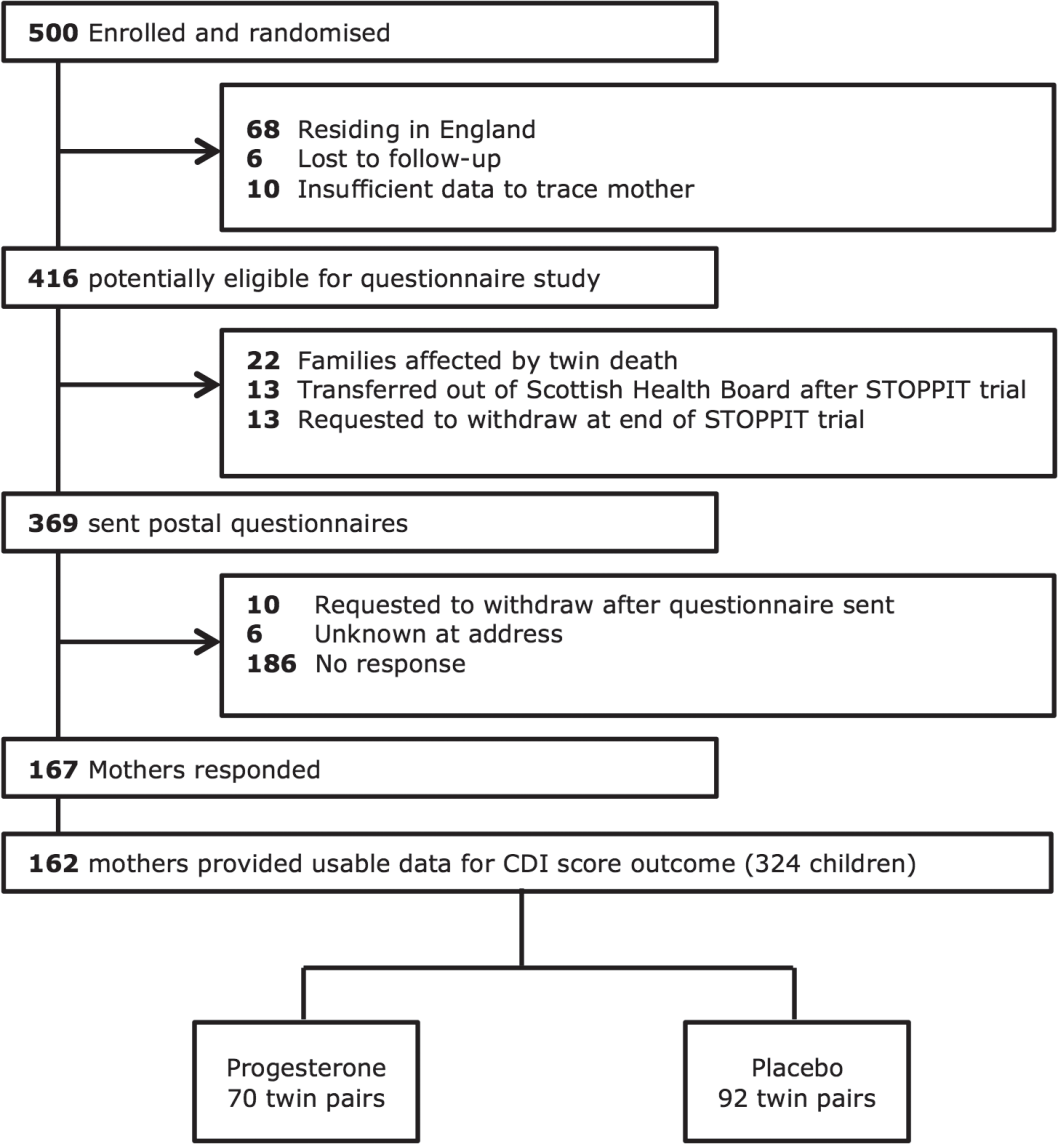
Flow of participants through questionnaire arm of study.

The flow of participants through the questionnaire arm of the study is shown in Fig 2. After relevant exclusions, 369 families were sent a questionnaire. A positive response was received from 167 mothers. Data for the primary outcome (CDI and HUI scores) were obtained from 162 mothers (44% of those mailed questionnaires).

The mean age of children at the time of questionnaire response was 55.5 months. S1 Table (see Supporting Information) shows the characteristics of the responders in comparison with the non-responders and those who were not sent a questionnaire. Responders tended to be older, had less social deprivation, had taken more study medication (progesterone and placebo), and their children were born at a higher gestational age. Within the group of responders, there were no differences in demographic characteristics when those allocated to progesterone were compared to those allocated to placebo. Additionally, within those who responded, there were no differences in demographic characteristics evaluated in those whose mothers were originally in the progesterone group compared with those whose mothers were originally in the placebo group.

Table 4 shows the proportion of children classified as having borderline or delayed development on the CDI. No significant difference in risk of abnormal development was seen between children who received progesterone and those who received placebo.

**Table 4:** Child Development Inventory score categorisation.

	**Progesterone**	**Placebo**	
	n/N children (%)	n/N children (%)	OR (95% CI), p-value
**Borderline development**	18/140 (13)	39/184 (21)	0.55 (0.26–1.19), 0.13
(25 to <30% below age range)			
**Delayed development**	42/140 (30)	65/184 (35)	0.87 (0.46–1.63), 0.66
(≥30% below age range)			
**Borderline/delayed development**	60/140 (43)	104/184 (57)	0.67 (0.35–1.28), 0.23
(≥25% below age range)			

The CDI is normed so that a child who performs at the level of a child that is ≥30% younger than their chronological age is classed as having delayed development. This equates to a developmental performance ≥2.0 standard deviations below the mean. Around 2% of children would be expected to be in this category. A child that performs at a level of a child 25 to <30% younger is between 1.5 and <3.0 SD below the mean for developmental performance and is classed as having borderline development. Around a further 3% of children would be expected to be in this category. An odds ratio less than one indicates a beneficial effect of progesterone.

Table 5 shows global health status in the progesterone and placebo groups. There were no differences in the global health rating or the individual multi-attribute health status (HUI Mark II and HUI Mark III) for children in the progesterone group compared with those in the placebo group.

**Table 5:** Health Utilities Index multi-attribute health status and global health rating.

	**Progesterone**	**Placebo**	
	Mean (SD), *n*	Mean (SD), *n*	Mean difference
			[95% CI], p-value
**Multi-attribute health status**			
HUI Mark II	0.96 (0.10), 147	0.96 (0.09), 184	0.00 [-0.03, 0.02], 0.70
HUI Mark III	0.96 (0.12), 147	0.97 (0.07), 184	-0.01 [-0.03, 0.02], 0.57
	**n/N children (%)**	**n/N children (%)**	**p-value**
**Global health rating**			
Excellent	129/147 (88)	166/184 (90)	0.51
Very good	14/147 (10)	14/184 (8)	
Good	4/147 (3)	4/184 (2)	

The Health Utilities Index includes two complementary systems of classification of multi-attribute health status in children. HUI Mark II measures health status as a function of seven attributes: sensation, ambulation, self-care, cognition, emotion, pain and fertility. The HUI Mark III assesses eight attributes: vision, hearing, speech, ambulation, dexterity, cognition, emotion and pain. Parental responses to questionnaire items are combined and ascribed a multi-attribute utility score between 0.00 and 1.00 (where 1.00 describes perfect health), calculated using standard formulas for the HUI Mark II and for the HUI Mark III. Additionally, parents give a global rating of child health on a 5-point Likert-type scale (excellent, very good, good, fair, poor).

The proportion of children in each group with a health utilities index of more than one (indicating some level of impairment) in each developmental domain is shown in S2 Table. Again, there were no differences in the individual components of the HUI score between the groups.

## Discussion

The results of this follow-up evaluation of children exposed *in utero* to progesterone for the prevention of preterm birth in women with twin pregnancies suggest that progesterone has no direct effect on child health and developmental outcomes. These findings complement the findings of the original STOPPIT trial in which progesterone was found to have no significant effect on the composite outcome of death or delivery before 34 weeks’ gestation [10].

The use of a cohort of women with twin pregnancies, in which progesterone had no effect on gestational length, allows the direct effect of progesterone to be determined independent of any effect of progesterone on preterm birth. Hence, our study provides no significant evidence that *in utero* progesterone exposure directly enhances the health of exposed children. This is perhaps surprising; progesterone has been shown to have anti-inflammatory and neuroprotective properties in models of adult acquired brain injury, and a direct beneficial effect on neurodevelopment is plausible [23]. In our study, the parent reported health status of twin children was excellent or very good for 98% of children, despite 33% of children having evidence of some developmental delay. The developmental delay likely relates to prematurity, with 28% of children having been born before 35 weeks’ gestation.

The results of this follow-up study additionally provide reassurance that progesterone does not cause harm to children exposed *in utero*. No difference was found in the overall rate of congenital anomalies in the progesterone and placebo groups. In the absence of an anomaly register, detection of congenital anomalies was limited to diagnoses made in children who presented to hospital. Nevertheless, anomalies that cause hospital admission and early death are likely to have been well recorded and linked.

Previous studies have suggested that progesterone exposure in early pregnancy might increase the risk of hypospadias in males [24]. In the STOPPIT trial, progesterone therapy was initiated after 24 weeks’ gestation. Thus, administration occurred after the completion of the embryological development of the male genitalia at 14 weeks. Current evidence indicates there is no increase in the risk of hypospadias in males exposed to progesterone after 16 weeks [25]. The results of our follow-up study support this hypothesis.

Other studies have raised concerns regarding behavioural outcomes in children exposed *in utero* to progesterone [26]. We reported no significant differences between the progesterone and placebo groups with respect to social development (reported on the CDI), emotion and cognition (reported on the HUI).

This follow-up did not include a formal evaluation of intelligence and school performance. Future linkage to educational records could demonstrate whether the apparently high incidence of some developmental delay has any adverse effects on educational attainment.

Our study extends the duration of follow-up of a twin cohort exposed to progesterone *in utero* to a mean age of 55.5 months. To date, limited evidence has been published regarding the long-term (post neonatal) effects of exposure *in utero* to vaginal progesterone in twins. Only one published study has evaluated the effects of vaginal progesterone. The previously published study had a larger sample size (n = 991), but the duration of postnatal follow-up was 18 months [12]. The duration of follow-up in our study is comparable with that in a study of intrauterine exposure to an alternative formulation of progesterone, 17 α-hydroxyprogesterone caproate [17]. This latter study of 274 singleton children, born to mothers participating in a randomised placebo-controlled trial of 17 α-hydroxyprogesterone caproate in high-risk singleton pregnancies, demonstrated no difference in child health measures despite prolongation of pregnancy (Table 6).

**Table 6:** Studies examining the long-term effects of prophylactic progesterone exposure *in utero* on childhood outcomes.

Author, year	Study population	Progesterone	Gestational age given	Delivery <34 weeks	Follow-up rate	Follow-up age	Congenital anomalies	Assessment tool 1 Progesterone vs. placebo	Assessment tool 2 Progesterone vs. placebo
**Northen *et al*** 2007 USA	Singletons (prior PTB)	Intramuscular 17 α-OHPC 250 mg/week	16–20 weeks to 36 weeks	RR 0.67 (0.48–0.93)^#^	80%	Mean 48 months	Genital / reproductive (2.1% vs. 1.2%; p = 1.0)	ASQ score below cut-off on at least one area (27.5% vs. 28%; p = 0.92)	PAI mean score (Boys: 66.5 vs. 67.3; p = 0.3. Girls: 32 vs. 33; p = 0.5)
**Rode *et al*** 2011 Austria/Denmark (PREDICT)	Twins	Vaginal natural progesterone 200 mg/day	20–24 weeks to 34 weeks	OR 0.80 (0.5–1.2)	79.2% and 74.8%*	6 months, 18 months	Congenital / chromosomal (3.8% vs. 4.0%; OR 1.0, 0.5–1.7)	ASQ mean score at 6 months of age (215 vs. 218; p = 0.45)	ASQ mean score at 18 months of age (193 vs. 194; p = 0.89)
**McNamara *et al*** 2015 United Kingdom (STOPPIT)	Twins	Vaginal natural progesterone 90 mg/day	24 to 34 weeks	OR 1.36 (0.89–2.09)	97% and 44%**	Mean 55 months	Congenital (4.0% vs. 4.0%; p = 0.92)	CDI score below cut-off on at least one area (30% vs. 35%; p = 0.66)	HUI global health rating‘Excellent’ (88% vs. 90%; p = 0.51)

PTB = preterm birth. 17 α -OHPC = 17 α -hydroxyprogesterone caproate. RR = relative risk (95% confidence interval). OR = odds ratio (95% confidence interval).

ASQ = Ages and Stages Questionnaire. PAI = Preschool Activities Inventory. CDI = Child Development Inventory. HUI = Health Utilities Index.

^#^Delivery <35 weeks’ gestation.

*In the PREDICT study, follow-up was achieved for 79.2% of twins at 6 months of age and 74.8% of twins at 18 months of age.

**In our study, follow-up was achieved for 97% of twins via record linkage of at least one health record and 44% of twins via parental questionnaire.

Given that the accuracy and reliability of paediatric developmental assessment is improved over time, the longer-term follow-up in our study is important. The relative neuroplasticity of the immature brain limits the capacity to ascertain a child’s neurodevelopmental status and prognosis until the age of at least two years [27]. Moreover, subtle delays are more easily identified as children become social and are exposed to environments where they are directly compared to peers [28]. This is particularly relevant where parent report is used as the method of assessment.

The rigour of our findings is enhanced by the use of record linkage, which avoids the selection bias inherent in questionnaire-based studies. Our ability to trace records on 97% of eligible children using record linkage demonstrates that in the Scottish population, record linkage is an economical and effective strategy for follow-up of children of women recruited to clinical trials in pregnancy. We believe that it is unlikely that lack of linkage to the 3% of missing children would have altered the overall findings.

Questionnaire data supported data from the record linkage and provided more detail on specific individual outcomes. However, the success rate of questionnaire follow-up was lower, with only 44% of eligible parents responding. Nevertheless, the questionnaire data supported the findings of our record linkage study (where the percentage of women and children followed up was excellent); exposure *in utero* to progesterone after 20 weeks’ gestation had no adverse effects.

The power of our follow-up study was dictated by the original sample of the STOPPIT trial. Additionally, the effective sample size of the follow-up study was reduced by the non-independence of data collected for each twin. Clustering was accounted for with multi-level modelling in our statistical analysis. If the impact of clustering is ignored, our study had 80% power to detect an absolute increase of 10% in hospital admissions, and 95% power to detect an absolute increase of 13% in hospital admissions in the progesterone group compared with the placebo group.

## Conclusions

In conclusion, we have found that exposure *in utero* to progesterone, given in twin pregnancies for the prevention of preterm birth, has no significant impact on child health and developmental outcomes at three to six years. We demonstrate that, in the absence of an effect on gestational length, progesterone has no beneficial or adverse effects on childhood development. The limitations of our study are acknowledged and the strengths asserted.

In women with high-risk singleton pregnancies because of short cervix or preterm delivery, progesterone administration (vaginal progesterone or intramuscular 17 α-hydroxyprogesterone caproate respectively) has been shown to reduce the rate of preterm birth. An ongoing randomised trial (OPPTIMUM, ISRCTN14568373) will determine whether vaginal progesterone administration to women at risk because of either a previous preterm birth or a short cervix is associated with long-term gains in terms of child health and development.

## Supporting Information

S1 Table Demographic characteristics of responders and non-responders and those not sent a follow-up questionnaire. (DOCX)

S2 Table Health Utilities Index individual domains. (DOCX)
