# Supplementary material for: STOPPIT Baby Follow-Up Study: The Effect of Prophylactic Progesterone in Twin Pregnancy on Childhood Outcome
Source: PLoS One. 2015 Apr 16;10(4):e0122341. doi: 10.1371/journal.pone.0122341 (PMC4400139; doi:10.1371/journal.pone.0122341)
Supplement: S1 Table — (DOCX) [file pone.0122341.s001.docx]

**S1 Table. Demographic characteristics of responders and non-responders and those not sent a follow-up questionnaire**

|  | Responders | | | Non-responders/Not eligible | | |  |
| --- | --- | --- | --- | --- | --- | --- | --- |
|  | **PR** | **Placebo** | **Total** | **PR** | **Placebo** | **Total** |  |
|  | **n/N children (%)** | | | **n/N children (%)** | | | **OR [95% CI], p-value** |
| Household index of multiple deprivation* |  |  |  |  |  |  |  |
| First quintile (most deprived) | 4/75 (5) | 11/92 (12) | 15/167 (9) | 25/134 (19) | 15/116 (13) | 40/250 (16) | 0.5 [0.3, 1.0], 0.04 |
| Second quintile | 9/75 (12) | 9/92 (10) | 18/167 (11) | 20/134 (15) | 22/116 (19) | 42/250 (17) | 0.6 [0.3, 1.1], 0.09 |
| Third quintile | 13/75 (17) | 11/92 (12) | 24/167 (14) | 18/134 (13) | 23/116 (20) | 41/250 (16) | 0.8 [0.4, 1.4], 0.41 |
| Fourth quintile | 22/75 (29) | 29/92 (32) | 51/167 (31) | 27/134 (20) | 22/116 (19) | 49/250 (20) | 1.4 [0.8, 2.3], 0.23 |
| Fifth quintile (least deprived) | 27/75 (36) | 32/92 (35) | 59/167 (35) | 44/134 (33) | 34/116 (29) | 78/250 (31) | 0.00 |
|  | **n/N children (%)** | | | **n/N children (%)** | | | **OR [95% CI],**  **p-value** |
| Chorionicity |  |  |  |  |  |  |  |
| Dichorionic | 62/75 (83) | 76/92 (83) | 138/167 (83) | 142/175 (81) | 128/158 (81) | 270/333 (81) | 1.1 [0.7, 1.8], 0.67 |
| Monochorionic | 13/75 (17) | 16/92 (17) | 29/167 (17) | 33/175 (19) | 33/158 (21) | 63/333 (19) | . |
|  | **Mean (SD), n** | | | **Mean (SD), n** | | | **Mean difference**  **[95%CI], p-value** |
| Maternal age at randomisation (years) | 35(4), 75 | 34(5), 92 | 34(5), 167 | 32(6), 175 | 32(6), 158 | 32(6), 333 | 2.2 [1.2, 3.2], <0.001 |
| Study drug doses administered | 60(19), 70 | 61(18), 90 | 60(19), 160 | 46(26), 155 | 47(26), 138 | 46(26), 293** | 13.9 [9.3,18.6], <0.001 |
| Twin gestational age at birth (weeks) | 36(3), 74 | 36(2), 91 | 36(3), 165 | 35(3), 172 | 35(3), 154 | 35(3), 326 | 0.8 [0.2, 1.3], 0.01 |
| Twin age at follow-up (months) | 55(10), 69 | 55(11), 91 | 55(11), 160 | 54(11), 112 | 54(12), 100 | 54(11), 212 | 0.9 [-1.3, 3.2], 0.42 |

* Scottish Index of Multiple Deprivation (SIMD) data are not available for women who were resident outwith Scotland at the time of study participation. The SIMD ranks the relative deprivation of 6005 population-defined ‘datazones’ across Scotland. Deprivation is estimated on the basis of datazone income (given a weight of 28%), employment (28%), health (14%), education (14%), access (9%), crime (5%) and housing (2%). The datazone and rank of each household are determined via postcode. Data are shown as the proportion of households falling into each quintile, where quintile one represents greatest deprivation and quintile five represents least deprivation.

** Women returned diaries indicating compliance with treatment. Some women did not complete or return their diary due to preterm delivery, advice from the treating team or because of incomplete compliance with treatment.
