# Supplementary material for: STOPPIT Baby Follow-Up Study: The Effect of Prophylactic Progesterone in Twin Pregnancy on Childhood Outcome
Source: PLoS One. 2015 Apr 16;10(4):e0122341. doi: 10.1371/journal.pone.0122341 (PMC4400139; doi:10.1371/journal.pone.0122341)
Supplement: S2 Table — (DOCX) [file pone.0122341.s002.docx]

**S2 Table. Health Utilities Index individual domains**

|  | Progesterone | Placebo |  |
| --- | --- | --- | --- |
|  | **n/N children (%)** | **n/N children (%)** | **p-value (Exact chi square)** |
| HUI Mark II |  |  |  |
| Sensation | 29/147 (20) | 44/184 (24) | 0.42 |
| Mobility | 1/147 (1) | 2/184 (1) | 1.00 |
| Emotion | 8/147 (5) | 9/184 (5) | 1.00 |
| Cognition | 6/147 (4) | 16/184 (9) | 0.12 |
| Self-care | 7/147 (5) | 8/184 (4) | 1.00 |
| Pain | 4/147 (3) | 10/184 (5) | 0.28 |
| Fertility | 0/147 (0) | 0/184 (0) | NA |
| HUI Mark III |  |  |  |
| Vision | 13/147 (9) | 26/184 (14) | 0.17 |
| Hearing | 0/147 (0) | 1/184 (1) | 1.00 |
| Speech | 22/147 (15) | 24/184 (13) | 0.63 |
| Ambulation | 1/147 (1) | 1/184 (1) | 1.00 |
| Dexterity | 1/147 (1) | 2/184 (1) | 1.00 |
| Emotion | 3/147 (2) | 6/184 (3) | 0.74 |
| Cognition | 7/147 (5) | 14/184 (8) | 0.37 |
| Pain | 5/147 (3) | 11/184 (6) | 0.31 |

Parental responses to questionnaire items are assigned a ‘level’ describing functional capacity (from optimal (1) to severely impaired (5-6)). The number of children with a HUI ‘level’ of greater than one (indicating some level of impairment) is shown for each domain.
